# Characterization of a new *Pseudomonas aeruginosa* Queuovirinae bacteriophage

**DOI:** 10.1128/spectrum.03719-23

**Published:** 2024-02-12

**Authors:** Lauren E. Whiteley, Marvin Whiteley

**Affiliations:** 1 School of Biological Sciences, Center for Microbial Dynamics and Infection, Georgia Institute of Technology, Atlanta, Georgia, USA; 2 Emory-Children’s Cystic Fibrosis Center, Atlanta, Georgia, USA; University of Sao Paulo, Sao Paulo, Brazil

**Keywords:** *Pseudomonas aeruginosa*, bacteriophage, Queuovirinae, pili

## Abstract

The ESKAPEE pathogen *Pseudomonas aeruginosa* is a common cause of chronic wound and cystic fibrosis lung infections, as well as acute burn and nosocomial infections. Many of these infections are recalcitrant to conventional antibiotic therapies due to both traditional antibiotic resistance mechanisms and antimicrobial tolerance. Recent successes with bacteriophage (phage) therapy to treat chronic human *P. aeruginosa* infections have led to a renewed interest in isolating and characterizing new *P. aeruginosa* phages. Here, we isolated and characterized a new lytic phage (termed PIP, pili-infecting phage) capable of infecting *P. aeruginosa* PA14. PIP is a tailed phage with an icosahedral head and flexible tail containing a genome that is 57,462 bp in length. Phylogenetic analysis reveals that PIP belongs to the subfamily Queuovirinae and genus Nipunavirus but is highly divergent in gene content from known Nipunaviruses. By isolating and characterizing a *P. aeruginosa* strain that spontaneously evolved resistance to PIP, we show that the receptor for PIP is Type IV pili. In summary, we isolated a new *P. aeruginosa* phage species with a unique genome, thus increasing the diversity of phages known to infect this important human pathogen.

**IMPORTANCE:**

The opportunistic pathogen *Pseudomonas aeruginosa* causes both acute and chronic human infections. These infections are notoriously difficult to treat due to both antibiotic resistance and antibiotic tolerance. The increasing frequency of antibiotic failure in *P. aeruginosa* infections has led scientists to explore other treatment options, including bacteriophage (phage) therapy. To this end, there has been a significant effort to identify new *Pseudomonas* phages. Here, we isolated and characterized a bacteriophage (termed PIP, pili-infecting phage) that infects *P. aeruginosa* PA14. Examination of the PIP genome revealed that this phage represents a new species in the subclass Queuovirinae. The isolation and characterization of spontaneous PA14 mutants that are resistant to PIP infection revealed Type IV pili as the PIP receptor. Ultimately, this study characterizes a new species of *Pseudomonas* phage, thus enhancing the known diversity of phages that infect this important pathogen.

## OBSERVATION

Bacteriophages (phages) are the most common biological entities on earth, found in any environment where bacteria exist. Yet despite their abundance, our knowledge about phages is far from extensive, and they have been deemed the “dark matter of the biological world” (1–3). Not only do gaps in knowledge exist in the sheer number of phage species on earth but also in the mechanisms they use to infect bacteria.

A renewed interest in the use of phages for treating infections (4–6) has led to a surge in the identification and characterization of new phages. This is particularly true for the ESKAPEE pathogen *Pseudomonas aeruginosa*, and phage therapy has shown promise in treating *P. aeruginosa* human chronic infections (4, 7, 8). *P. aeruginosa* is a common cause of chronic wound and cystic fibrosis lung infections, as well as acute nosocomial infections (9). Phages provide a potential therapeutic solution to address the fact that many of these infections are not cleared with conventional antibiotic therapies due to both antibiotic resistance and tolerance mechanisms. Here, we isolated and characterized a new species of phage (termed PIP, pili-infecting phage) capable of infecting *P. aeruginosa* strain PA14.

PIP was isolated from a stream in Decatur, GA, USA in 2021, located at latitude 33.76 and longitude −84.29. This is an urban area, and the stream had been contaminated by raw sewage numerous times prior to our collection. Water and sediment were collected from the stream, filtered through a 0.45 µm pore membrane, and then mixed with *P. aeruginosa* strain PA14 in soft agar to test for the presence of phage. Twelve plaques were observed after 24 hours. Plaques were 1–2 mm in diameter and mostly clear with observable halos. A single plaque was picked, the phage was amplified in PA14 planktonic cells overnight, and then the plaque was purified to yield a pure culture of PIP.

**Fig 1:**
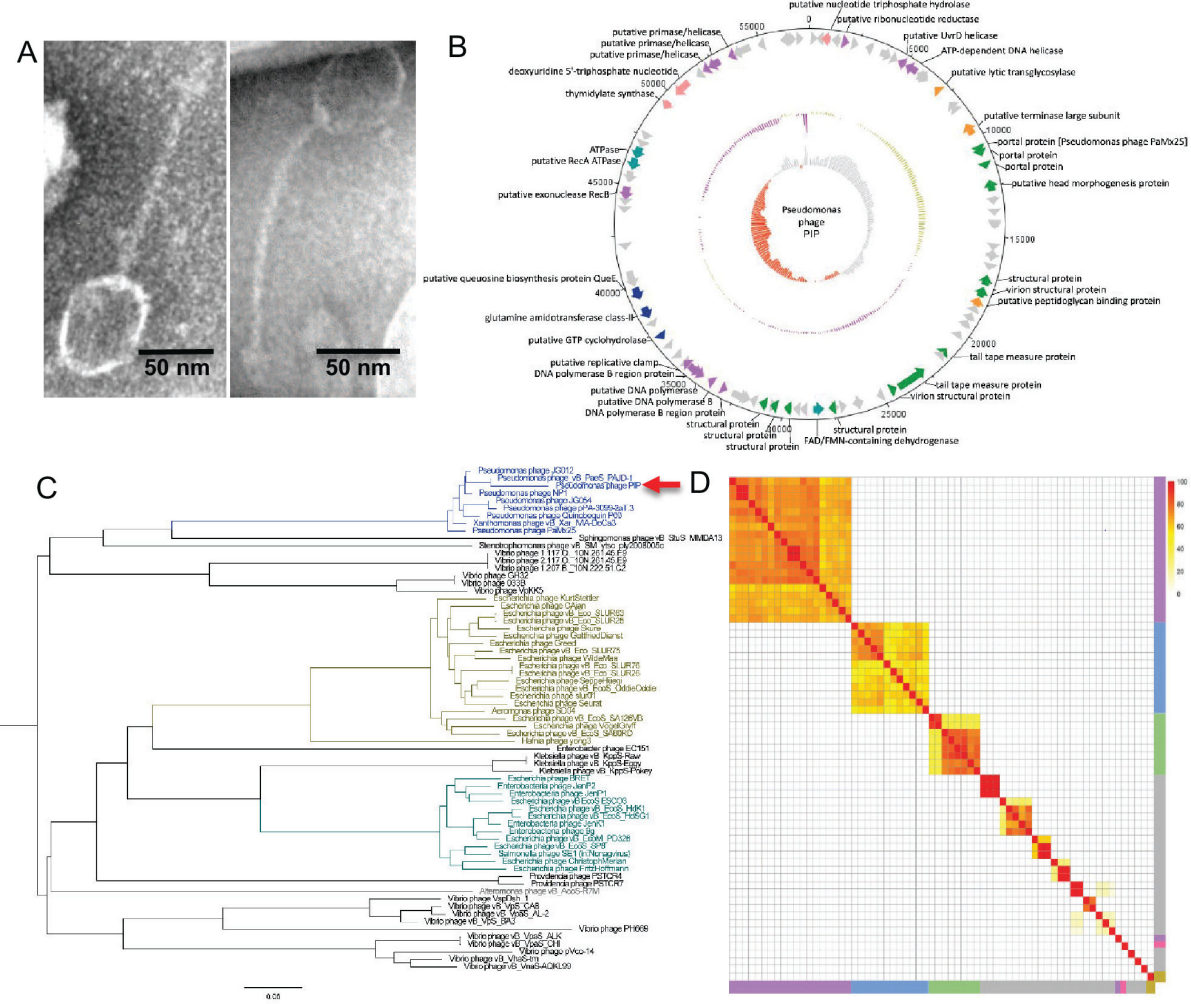
Characterization of *Pseudomonas* phage PIP. (A) Negative stain TEM images of PIP. (**B)** PIP genome map. Open reading frame functions were sorted into eight classes: nucleotide metabolism and transport (pink), DNA replication and repair (purple), lysis (orange), structural (green), energy production and conversion (light blue), biosynthesis (dark blue), and hypothetical proteins (gray). (**C)** Phylogenetic relationships of all available Queuovirinae viruses constructed with VICTOR and visualized with FigTree. The red arrow points to PIP. (**D)** Heatmap of the percentage of proteins shared between *Pseudomonas* Queuovirinae phage genomes. The four genera of Queuovirinae were highlighted along with unclassified Queuovirinae including Seuratvirus (purple), Nonagvirus (blue), Nipunavirus (green), Amoyvirus (yellow), and unclassified (gray). PIP is highlighted in pink. The scale bar on the top right is the percentage of shared proteins between two phages. All Queuovirinae genomes for these analyses were downloaded on 6 June 2023.

Examination of PIP using negative-stain transmission electron microscopy revealed a tailed phage with an icosahedral head and flexible tail (Fig. 1A). This physical structure, according to the guidelines set forth by the International Committee on Taxonomy of Viruses (ICTV), is characteristic of the Caudoviricetes class of phages. PIP has a capsid size of 59.3 ± 3.6 nm in diameter and a tail length of 120.2 ± 4.4 nm (Fig. 1A).

We sequenced the genome of PIP using Nanopore long-read sequencing (970× coverage), yielding a double-stranded DNA genome 57,462 bp in length with a GC content of 57%, which is lower than that in *P. aeruginosa* PA14 (66%). Using Prokka (10), we identified 90 predicted open reading frames (ORFs) in the PIP genome (Fig. 1B). BLASTX was performed manually for each of the ORFs, revealing 37 PIP ORFs that had homology (E-value ≤ 1 × 10^−5^) to proteins with known function (Table S1). The remaining genes were designated as hypothetical. Most of the PIP ORFs had homology to other *Pseudomonas* phages belonging to the subfamily Queuovirinae and genus Nipunavirus. PIP is categorized as a lytic phage as genes commonly associated with lysogeny, including an integrase and an excisionase, were not identified on the genome.

A BLASTN search of the entire PIP sequence revealed that it had the highest sequence identity to the Nipunavirus *Pseudomonas* phage vB_PaeS_PAJD-1, a phage put forth as a candidate for phage therapy against *P. aeruginosa* mastitis infection (11). Similarity analysis showed that PIP had a percent identity of 80.35% and a query cover of 91% when compared to phage vB_PaeS_PAJD-1. To further assess the taxonomy of PIP, a phylogenetic tree of all available Queuovirinae viruses (downloaded on 6 June 2023) was constructed with VICTOR (12) and visualized with FigTree. PIP clustered with Nipunavirus phages, with the closest related phage being vB_PaeS_PAJD-1 at a branch distance of 0.0623 (Fig. 1C). Based on these results, and the fact that ICTV guidelines classify a phage as a new species if it has less than 95% sequence identity to another phage (13), we propose PIP as a new *Pseudomonas* phage species.

To further examine the similarities between PIP and other related viruses, a pangenome was constructed for all Queuovirinae phages, yielding 2,062 genes. The pangenome had 0 core genes (genes included in 99%–100% of all Queuovirinae phages), 0 softcore genes (95%–99%), 115 shell genes (15%–95%), and 1,947 cloud genes (0%–15%). To examine the similarity in gene content between Queuovirinae, pairwise comparisons between all phage genomes were performed, and a heatmap was created showing the percentage of proteins shared between each pair of phages (Fig. 1D). This analysis revealed that an average of 17 genes were present in any two phages, and an average of 304 genes were present in only one of the phages in each pairwise comparison. Although most phages clustered by genus in the pairwise comparisons, both PIP and Hafnia phage yong3 did not. These data indicate that there are significant gene content differences in the Queuovirinae, and PIP is substantially different from other members of this subfamily and the Nipunavirus genus.

**Fig 2:**
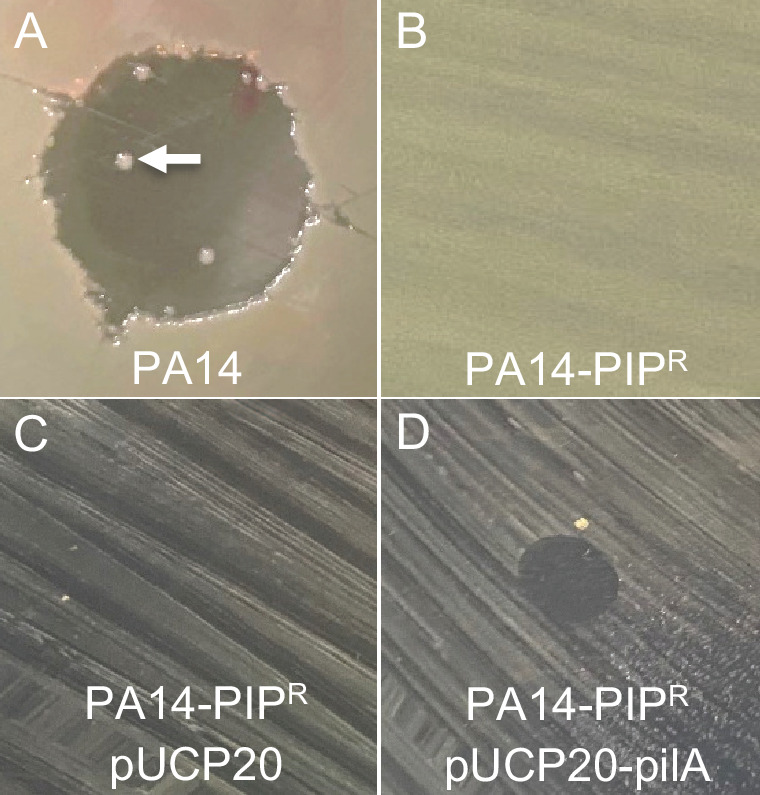
Type IV pili are required for PA14 PIP infection. (A) PIP zone of clearing on strain PA14 with resistant strains growing within the lysis zone (white arrow). (**B)** PIP causes no zone of clearing on the PA14 spontaneous resistance mutant PA14-PIP^R^. (**C)** PA14-PIP^R^ carrying the plasmid pUCP20 is resistant to PIP. (**D)** Introduction of the *pilB* complementation plasmid pUCP20-pilB into PA14-PIP^R^ restored sensitivity to PIP. Note: the size of the zones of clearing does not indicate relative susceptibilities, as the images in panels A and B were taken at a higher magnification to allow visualization of resistant colonies in panel A.

During our testing of the activity of our purified phage stocks, we observed PA14 colonies growing within the PIP lysis zone (Fig. 2A, white arrow). We isolated one of these colonies (termed PA14-PIP^R^) and confirmed that it was indeed resistant to PIP infection (Fig. 2B). We then sequenced PA14-PIP^R^ using Nanopore long read sequencing (223× coverage) and compared this sequence to that of PA14 to identify potential mutations responsible for PIP resistance. Single nucleotide polymorphism (SNP) analysis revealed 95 SNPs in PA14-PIP^R^ compared to our wild-type PA14 (Table S2), including an SNP at base 79 of the coding sequence of *pilB*, which resulted in a premature stop codon. This SNP results in a truncated PilB protein containing 26 amino acids instead of the normal 567 amino acids. PilB belongs to the secretion NTPase superfamily that powers the extension of Type IV pili in *P. aeruginosa*, and *pilB* mutants are unable to produce Type IV pili (14, 15). We confirmed a functional defect in Type IV pili in PA14-PIP^R^ as this strain showed a defect in twitching motility (Fig. S1A). Since Type IV pili are common receptors for *Pseudomonas* phage (16, 17), we hypothesized that this SNP was responsible for PIP resistance. To test this, we constructed a plasmid containing PA14 *pilB* under the control of its native promoter (pUCP20-pilB), then introduced this plasmid or its parent plasmid pUCP20 into PA14-PIP^R^ and tested for PIP susceptibility. As expected, PA14-PIP^R^ carrying pUCP20 was not susceptible to PIP (Fig. 2C). However, PA14-PIP^R^ carrying pUCP20-pilB rescued susceptibility to PIP (Fig. 2D), indicating that the mutation in *pilB* is responsible for the resistance of PA14-PIP^R^ to PIP infection. As expected, pUCP20-pilB also rescued twitching motility in PA14-PIP^R^ (Fig. S1A).

To examine whether PIP also infects other *P. aeruginosa* strains that produce Type IV pili, we tested the susceptibility of the laboratory strains PAO1, PAK, and 19SJ (18–20). While 19SJ was susceptible to PIP infection, both PAO1 and PAK were resistant (Fig. S1B), indicating that not all *P. aeruginosa* strains that produce Type IV pili are susceptible to PIP. This is likely due to either differences in the amino acid sequence of the pilin protein or differential pilin glycosylation.

In summary, we have isolated and characterized a new phage species for *Pseudomonas aeruginosa* with unique genomic content, thus increasing the known diversity of *Pseudomonas* phage.

## Data Availability

The raw sequencing files from this study can be found at NCBI Sequence Read Archive (SRA) under the accession number OR687155 for the phage genome and CP136842 for the PA14 pilB mutant.

## SUPPLEMENTAL MATERIAL

Supplemental material spectrum.03719-23-s0001.docx Fig. S1 and all experimental details.

Table S1 spectrum.03719-23-s0002.csv PIP genes with putative functions.

Table S2 spectrum.03719-23-s0003.csv SNPs identified in PA14-PIPR using medaka.

ASM does not own the copyrights to Supplemental Material that may be linked to, or accessed through, an article. The authors have granted ASM a non-exclusive, world-wide license to publish the Supplemental Material files. Please contact the corresponding author directly for reuse.
