# Supplementary material for: Characterization of a new Pseudomonas aeruginosa Queuovirinae bacteriophage
Source: Microbiol Spectr. 2024 Feb 12;12(3):e03719-23. doi: 10.1128/spectrum.03719-23 (PMC10913419; doi:10.1128/spectrum.03719-23)
Supplement: Supplemental material — Fig. S1 and all experimental details. [file spectrum.03719-23-s0001.docx]

**Supplementary Materials**

**A**


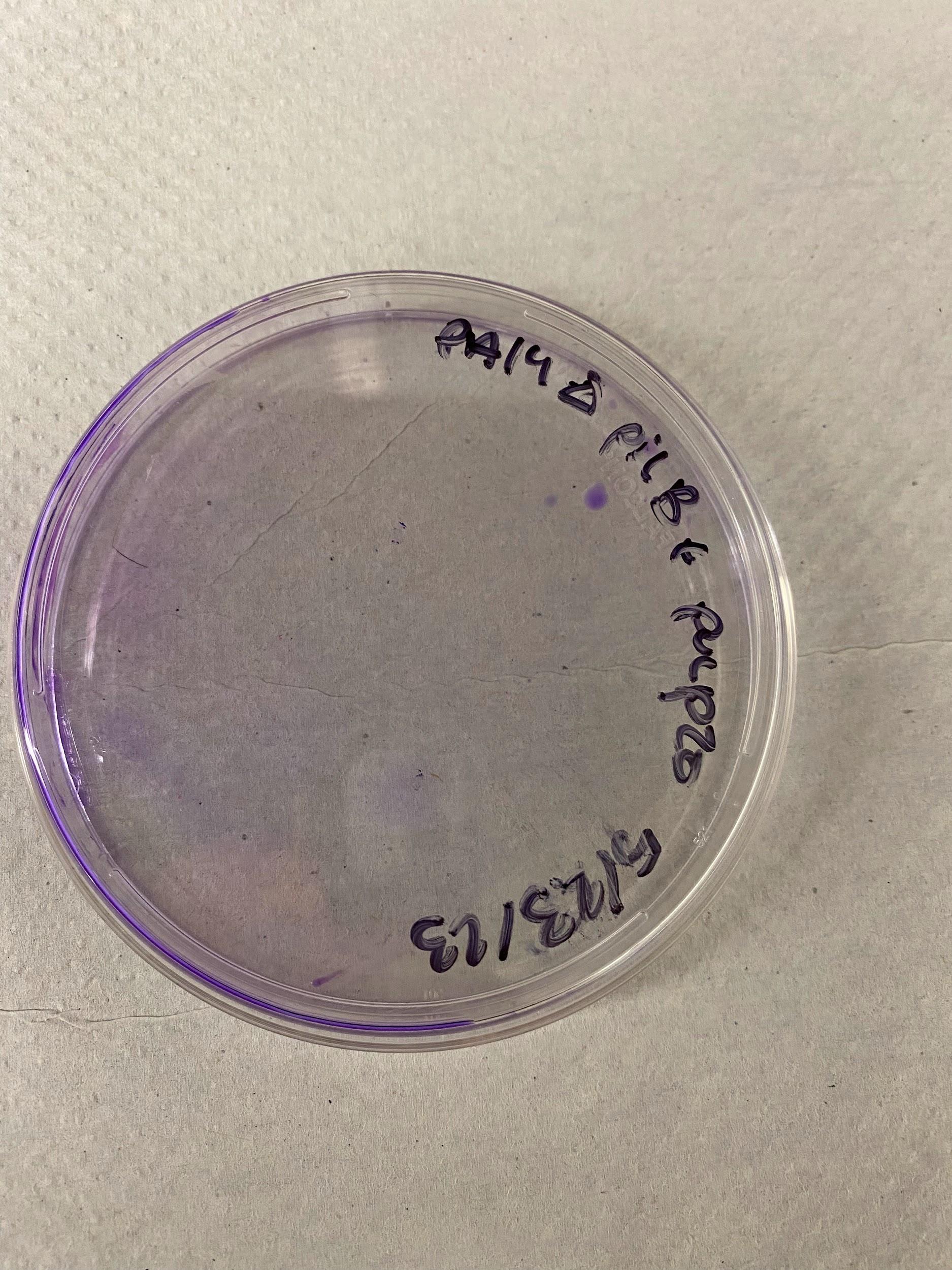

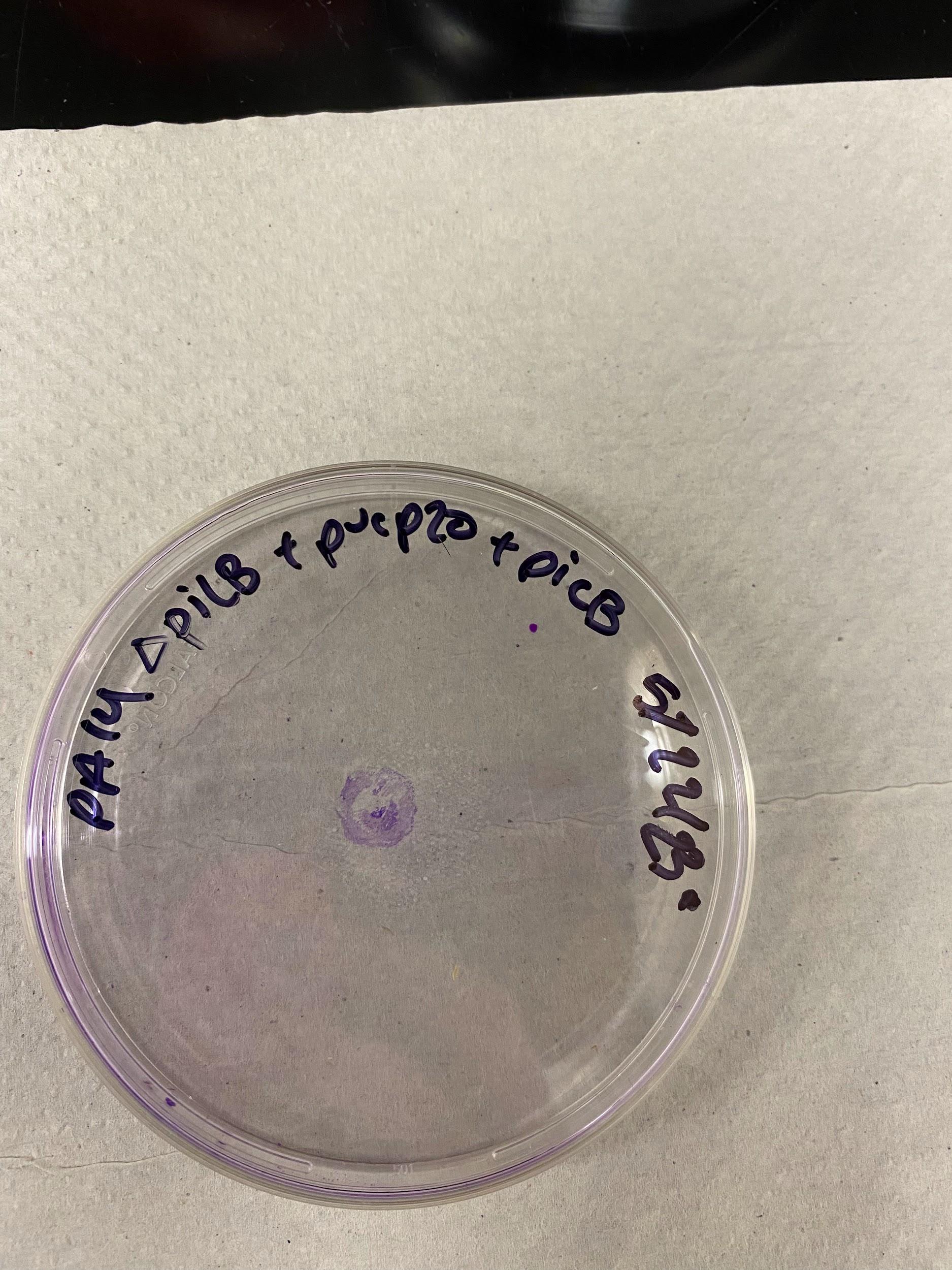


**PA14-PIP^R^**

**pUCP20-pilB**

**PA14-PIP^R^**

**pUCP20**


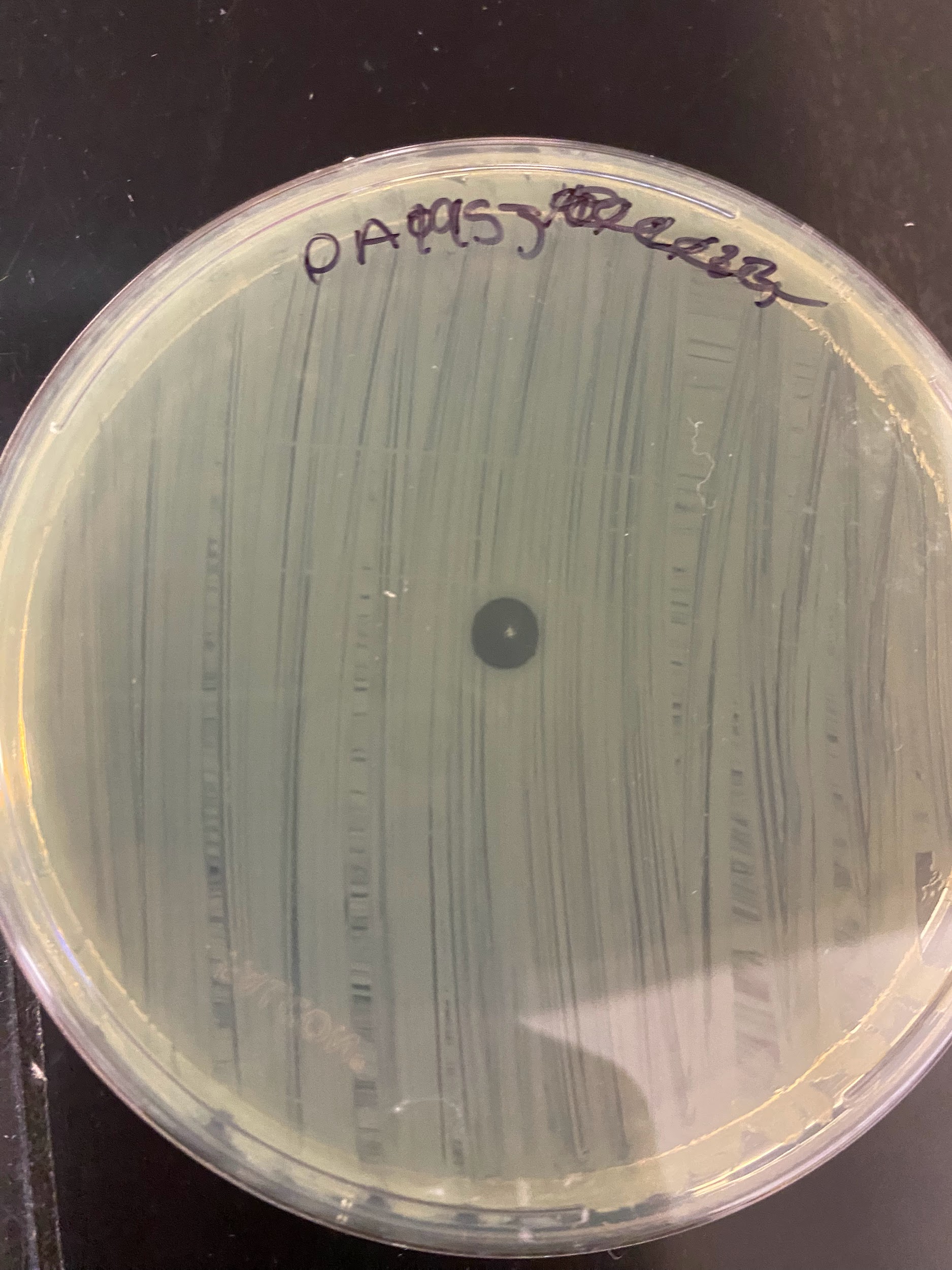

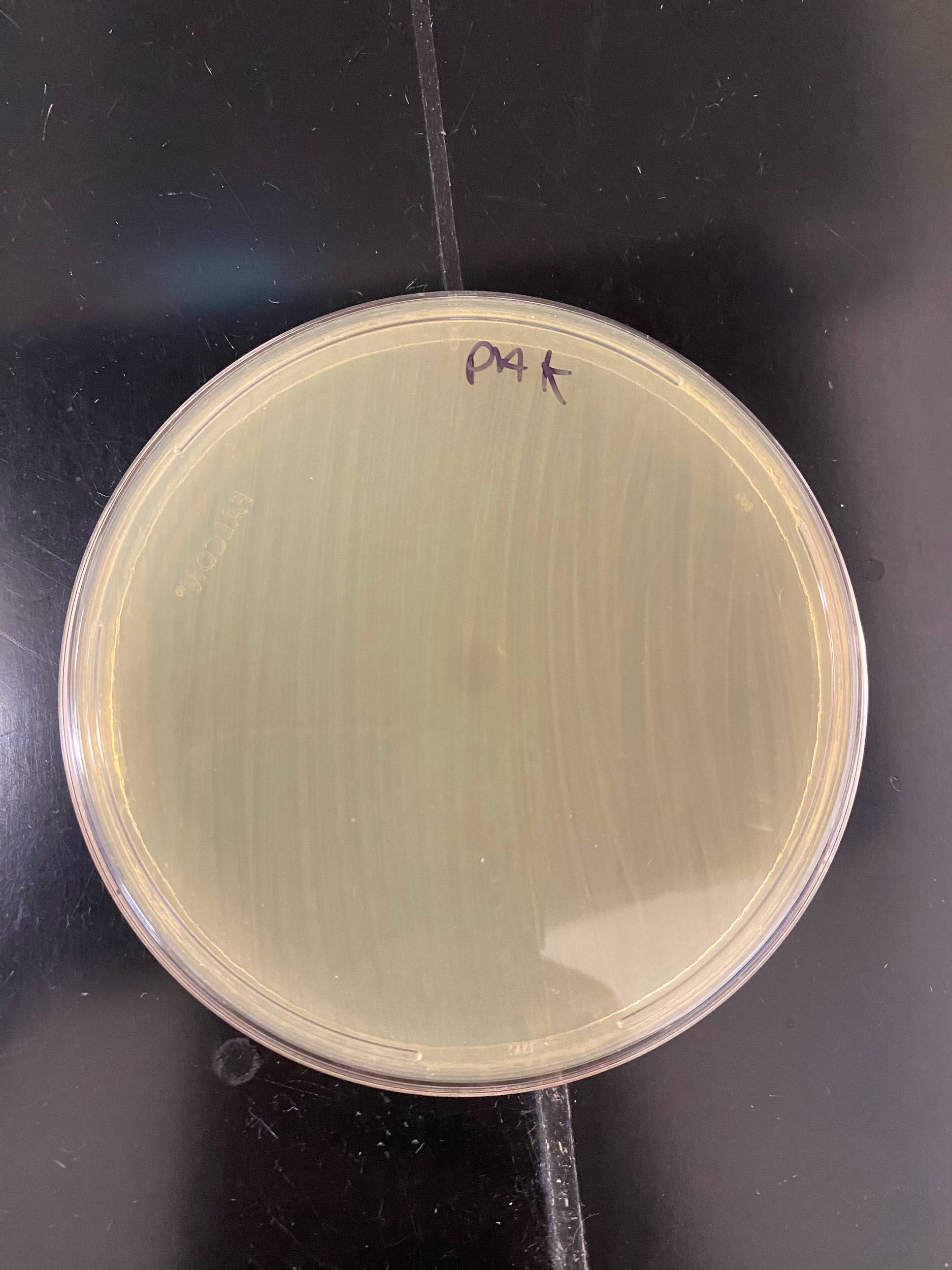

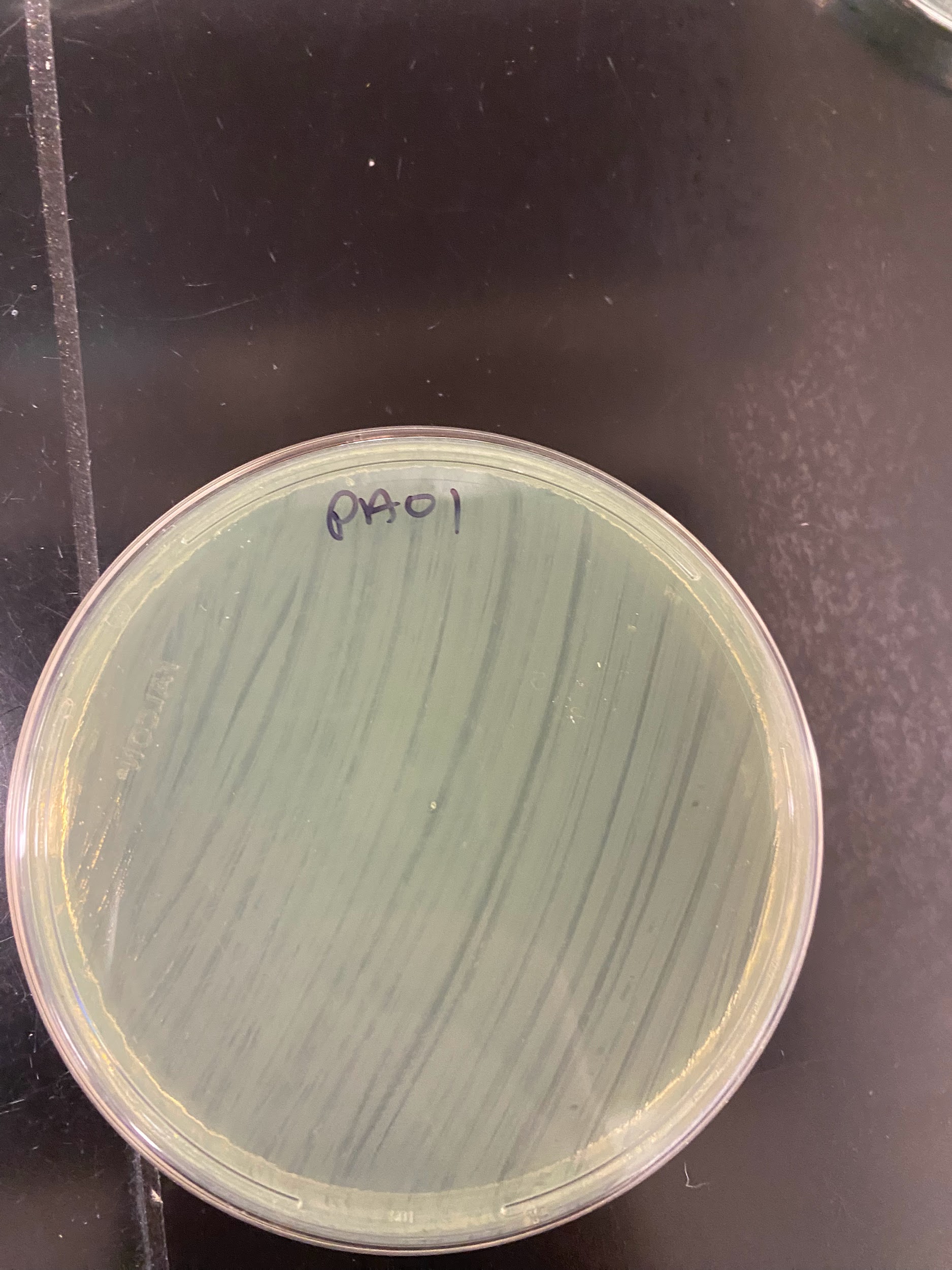

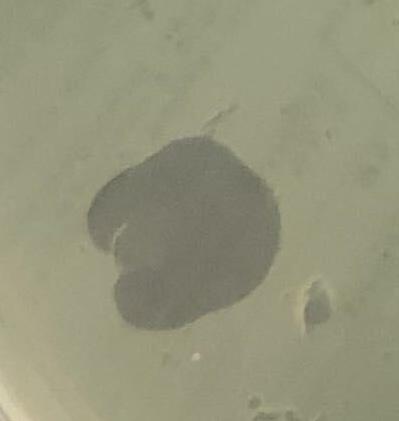


**B**

**Fig. S1.** **A.** Twitching motility assays of the spontaneous PIP resistant strain PA14-PIP^R^ carrying the cloning vector pUCP20 (left) or the complementation vector pUCP20-pilB (right). Shown are crystal violet-stained zones of twitching (not evident for PA14-PIP^R^ pUCP20) on the bottom of agar petri dishes. **B.** *P. aeruginosa* strains PA14 and 19SJ, but not PAO1 and PAK, are susceptible to PIP infection.

**19SJ**

**PAK**

**PAO1**

**PA14**

**Materials and Methods**

**Bacterial strains and culture conditions.** *Pseudomonas aeruginosa* strain PA14 (obtained from the Ausubel group, (1)) was used for phage isolation and propagation. *P. aeruginosa* strains mPAO1, PAK, and 19SJ were also used for PIP sensitivity studies (2-4). Unless otherwise specified, all cultures were incubated in Lysogeny Broth (LB) at 37°C shaking at 200 rpm. Antibiotics were added to the medium as necessary: 10 µg/ml ampicillin for *Escherichia coli* and 300 µg/ml carbenicillin for *P. aeruginosa*.

**Phage isolation and propagation.** *Pseudomonas* phage PIP was isolated from a small creek (latitude 33.76: longitude -84.29) located in Decatur, Georgia USA that was contaminated by raw sewage numerous times prior to our collection. A water sample was taken (10 mL) and centrifuged at 8500 x g for 10 minutes to remove large soil particles. The sample was then filter sterilized through a 0.45 μm syringe filter. For phage isolation, 500 µl of the filtered water sample was added to 4.5 mL of exponentially-growing PA14. Samples were vortexed and incubated for 30 minutes at 25°C. Following incubation, 500 µl of the bacterial/water sample was added to 3 ml of 0.7% melted LB agar at 55˚C, then immediately poured on top of LB agar plates. Agar was allowed to solidify, and plates were incubated overnight at 37°C at which point plaques were observed. To propagate the phage, a sterile p100 pipette tip was used to remove a single plaque, the plaque was added to 4.5 ml of LB containing exponentially-growing PA14, incubated overnight at 37°C, then bacteria removed from the phage/bacterial culture by centrifugation at 16,000 x g for five minutes. The supernatant was transferred to a clean tube with 100 μl of chloroform, vortexed for 30 seconds, then centrifuged at 16,000 x g for five minutes. The top layer of the sample was transferred to a clean tube and stored at 4°C. This procedure was performed two times, each time taking an individual phage plaque.

**Phage quantification and infection.** Plaque Forming Unit (PFU) assays were performed to quantify phage numbers as described (5) using LB agar. Plaque size was measured with Image J (6). To assess PIP infection of *P. aeruginosa* strains, 5 µl of PIP stocks was spotted onto the top of LB agar plates that had been swabbed with an overnight culture of *P. aeruginosa*.

**Transmission Electron Microscopy.** PIP morphology was examined by negative stain transmission electron microscopy (TEM). Concentrated PIP (10^9^ PFU/mL) was stained with phosphotungstic acid as described (7) and imaged using JEOL 100CX II electron microscope (JEOL Ltd. Tokyo, Japan). Phage measurements were taken with ImageJ (6).

**Phage DNA extraction, sequencing, and annotation.** Phage and bacterial DNA was extracted using the DNeasy PowerSoil Pro Kit (Qiagen). DNA concentrations were quantified with Nanodrop. Genomes were sequenced with Nanopore long-read sequencing at Seqcenter (Pittsburgh, Pennsylvania USA). According to NanoPlot v.1.42.0 (17), the phage genome had 49,451 reads, with a mean read quality of 7.4. The PA14 PIP-resistant strain had 97,113 reads with an average read quality of 12.6. Wildtype PA14 had 134,154 reads with an average read quality of 10.5. Fastq files were assembled using Flye version 2.9.1 with default parameters (8) and polished with medaka version 1.7.0 with default parameters (https://github.com/nanoporetech/medaka).

The phage genome ORFs were identified with Prokka v.1.14.5 through KBase with options adjusted to viral kingdom. The called ORFs were blasted on NCBI and annotations with an E cutoff value of 1 × 10^−5^ were used Phage genome annotations for the heatmap were performed with Prokka (9) using a custom ‘*Caudoviricetes*’ database. The database was created by concatenating Genbank files of Caudoviricetes genomes downloaded from NCBI (<https://www.ncbi.nlm.nih.gov/nuccore>). The remaining ORFs were manually annotated using BLASTn from NCBI (<https://www.ncbi.nlm.nih.gov/BLAST/>). The resulting genome was visualized using Artemis dna plotter version 18.2 (10). SNP analysis was accomplished with medaka version 1.6.1 with default parameters.

**Phylogenic analysis.** The Virus Classification and Tree Building Online Resource (VICTOR) was used to calculate genome-to-genome distance of green phage and other phages in the subfamily Queuovirinae (11). The Genome-BLAST Distance Phylogeny (GBDP) method was used for pairwise comparisons of the amino acid sequences including 100 pseudo-bootstrap replicates (12). Settings were optimized for prokaryotic viruses and whole nucleotide sequences, and the tree was rooted at the midpoint and visualized using FigTree version 1.4.4 (<http://tree.bio.ed.ac.uk/software/figtree/> and <https://github.com/rambaut/figtree/>).

**Pangenome analysis.** All *Pseudomonas* phage genomes were downloaded from the NCBI nucleotide database (<https://www.ncbi.nlm.nih.gov/nuccore>) in the FASTA file format. Genomes were reannotated with Prokka (9) using a custom PHROGs database developed by Andrew Millard’s lab at University of Leicester (<http://millardlab.org/2021/12/15/inphared-re-annotated-with-phrogs/>). Roary (13) was used for pangenome analysis, and results were further processed using Roary-specific analysis scripts (<https://github.com/rehrlich/roary_analysis>). A heatmap was created from the results, and visualized with R using the complete linkage method (14).

**Twitching assay.** Twitching assays were performed as described (15) using LB agar plates and staining with 1% wt/volume crystal violet.

**Construction of the *pilB* complementation plasmid.** For complementation experiments, pUCP20 was used. *P. aeruginosa* PA14 *pilB* was amplified from chromosomal DNA using Phusion High-Fidelity DNA Polymerase as per manufacturer’s instructions (New England Biolabs) with the primers

5’-CGACGGCCAGTGCCATCCGACCTTCTCGGTTTG-3’

5’-AGCTCGGTACCCGGGTTAGTCCTTGGTCACGCG-3’.

pUCP20 and the *pilB* amplicon were assembled using the Gibson Assembly Cloning kit (NEB), and the resultant plasmid (pUCP20-pilB) was transformed into electrocompetent *Escherichia coli* and verified using DNA sequencing. Plasmids were electroporated into *P. aeruginosa* as described (16).

**References**

1. Liberati NT, Urbach JM, Miyata S, Lee DG, Drenkard E, Wu G, Villanueva J, Wei T, Ausubel FM. 2006. An ordered, nonredundant library of *Pseudomonas aeruginosa* strain PA14 transposon insertion mutants. Proc Natl Acad Sci USA 103:2833-8.

2. Deziel E, Paquette G, Villemur R, Lepine F, Bisaillon J. 1996. Biosurfactant production by a soil pseudomonas strain growing on polycyclic aromatic hydrocarbons. Appl Environ Microbiol 62:1908-12.

3. Jacobs MA, Alwood A, Thaipisuttikul I, Spencer D, Haugen E, Ernst S, Will O, Kaul R, Raymond C, Levy R, Chun-Rong L, Guenthner D, Bovee D, Olson MV, Manoil C. 2003. Comprehensive transposon mutant library of *Pseudomonas aeruginosa*. Proc Natl Acad Sci USA 100:14339-44.

4. Takeya K, Amako K. 1966. A rod-shaped *Pseudomonas* phage. Virology 28:163-5.

5. Panec M, Katz D. 2006. Plaque assay protocol. Protocols American Society for Microbiology, Washington, DC: <https://asmorg/Protocols/Plaque-Assay-Protocols>.

6. Schneider CA, Rasband WS, Eliceiri KW. 2012. NIH Image to ImageJ: 25 years of image analysis. Nat Methods 9:671-5.

7. Mashburn LM, Whiteley M. 2005. Membrane vesicles traffic signals and facilitate group activities in a prokaryote. Nature 437:422-5.

8. Kolmogorov M, Yuan J, Lin Y, Pevzner PA. 2019. Assembly of long, error-prone reads using repeat graphs. Nat Biotechnol 37:540-546.

9. Seemann T. 2014. Prokka: rapid prokaryotic genome annotation. Bioinformatics 30:2068-9.

10. Carver T, Thomson N, Bleasby A, Berriman M, Parkhill J. 2009. DNAPlotter: circular and linear interactive genome visualization. Bioinformatics 25:119-20.

11. Meier-Kolthoff JP, Goker M. 2017. VICTOR: genome-based phylogeny and classification of prokaryotic viruses. Bioinformatics 33:3396-3404.

12. Meier-Kolthoff JP, Auch AF, Klenk HP, Goker M. 2013. Genome sequence-based species delimitation with confidence intervals and improved distance functions. BMC Bioinformatics 14:60.

13. Page AJ, Cummins CA, Hunt M, Wong VK, Reuter S, Holden MT, Fookes M, Falush D, Keane JA, Parkhill J. 2015. Roary: rapid large-scale prokaryote pan genome analysis. Bioinformatics 31:3691-3.

14. Oliveira H, Domingues R, Evans B, Sutton JM, Adriaenssens EM, Turner D. 2022. Genomic Diversity of Bacteriophages Infecting the Genus *Acinetobacter*. Viruses 14.

15. Semmler AB, Whitchurch CB, Mattick JS. 1999. A re-examination of twitching motility in Pseudomonas aeruginosa. Microbiology (Reading) 145 ( Pt 10):2863-73.

16. Choi KH, Kumar A, Schweizer HP. 2006. A 10-min method for preparation of highly electrocompetent *Pseudomonas aeruginosa* cells: application for DNA fragment transfer between chromosomes and plasmid transformation. J Microbiol Methods 64:391-7.

17. Wouter De Coster, Rosa Rademakers, NanoPack2: population-scale evaluation of long-read sequencing data, *Bioinformatics*, Volume 39, Issue 5, May 2023, btad311, <https://doi.org/10.1093/bioinformatics/btad311>.
